# Personal

**Published:** 1914-10

**Authors:** 


### PERSONAL.

---

Announcement of removal, travel, and other matters of interest are requested. Please report errors in the listing of any physician in the State and other directories, that we may co-operate with the State Society in securing a correct list.

---

Dr. Leon Hamilton, of Elmira, spent several days during September visiting friends in Buffalo.

---

Dr. Edward E. Powers, U. B., 1912, of Rome, was married in Buffalo on the 9th of September.

---

Dr. John M. Garratt, of Buffalo, has been appointed a member of the consulting staff of the J. N. Adam Memorial Hospital at Perrysburg, and to the Tuberculosis Dispensary. He will have charge of the Department of Roentgenology.

---

Dr. John Middleton, of Buffalo, returned about the first of September from a trip of six weeks to Vancouver and the Northwest.

---

Dr. Lucius G. Waterman, of Buffalo, enjoyed a vacation on the Atlantic Coast in August and September.

---

Dr. Wm. A. Peart, of Sanborn, returned from Europe, September 1st.

---

Dr. Lucien Howe returned from Europe, via Liverpool, late in September.

---

Dr. C. A. Clements, of Buffalo, spent two weeks at Atlantic City in September.

Dr. C. J. Carr, of Buffalo, who was operated upon for appendicitis, July 2nd, returned the first of September from a trip to the Blue Ridge Mountains, Atlantic City and the Adirondacks, fully restored to health.

---

Dr. Frederick W. Burkhardt, of Buffalo, returned from Europe, September 7.

---

Dr. Rudolf C. Miller, Buffalo, 1909, formerly of Buffalo, is practicing in Monroe, Wash.

---

Dr. C. H. Andrews, of Buffalo, has been appointed by the Governor to represent New York State at the National Prisons Association Convention at St. Paul, October 3 to 8.

